# Glioblastoma Multiforme: An Overview of Emerging Therapeutic Targets

**DOI:** 10.3389/fonc.2019.00963

**Published:** 2019-09-26

**Authors:** Olivia G. Taylor, Joshua S. Brzozowski, Kathryn A. Skelding

**Affiliations:** ^1^Faculty of Health and Medicine, Priority Research Centre for Cancer Research, Innovation and Translation, School of Biomedical Sciences and Pharmacy, University of Newcastle, Callaghan, NSW, Australia; ^2^Hunter Cancer Research Alliance and Cancer Research Program, Hunter Medical Research Institute, New Lambton Heights, NSW, Australia

**Keywords:** glioblastoma, targeted therapeutics, anti-cancer drugs, brain cancer, immunotherapy

## Abstract

Glioblastoma multiforme (GBM) is the most common and aggressive malignant primary brain tumour in humans and has a very poor prognosis. The existing treatments have had limited success in increasing overall survival. Thus, identifying and understanding the key molecule(s) responsible for the malignant phenotype of GBM will yield new potential therapeutic targets. The treatment of brain tumours faces unique challenges, including the presence of the blood brain barrier (BBB), which limits the concentration of drugs that can reach the site of the tumour. Nevertheless, several promising treatments have been shown to cross the BBB and have shown promising pre-clinical results. This review will outline the status of several of these promising targeted therapies.

## Introduction

Glioblastoma multiforme (GBM) is the most common and aggressive primary malignant brain tumour and accounts for 60% of brain tumours in adults (1). The global incidence of GBM is <10 per 100,000 persons (2) and has increased over the last decade (3). GBM patients have a poor prognosis with a 1-year survival rate of 37.2%, a 5-year survival rate of 5.1% (4) and a median survival of ~10 months (5). GBM is divided into three subgroups based on isocitrate dehydrogenase 1 (IDH1) and IDH2 mutation status: IDH-mutant, IDH-wild-type and NOS (not otherwise specified) (6–8). However, despite this classification, the majority of GBM patients receive identical treatments, and few targeted therapies currently exist, contributing to the poor outcomes typically experienced by GBM patients. This review will outline the current treatment options for GBM and discuss some of the more recent developments in targeted therapies being investigated for the treatment of GBM.

## Current Treatment Options

The treatment of brain tumours faces unique challenges, most notably the presence of the blood brain barrier (BBB), a highly selective semipermeable barrier that separates blood from the brain. The BBB is comprised of the endothelial cells of capillaries, astrocytes surrounding the capillary, and pericytes embedded in the capillary basal lamina. Physiochemical properties including molecular weight, lipophilicity and charge affect the ability of a molecule to cross the BBB (9). The BBB prevents nearly all large molecules (>400 Da) (10) and ~98% of small molecule drugs from entering the central nervous system (CNS) (11). The current treatment pipeline begins with surgical resection of the tumour, if applicable and safe to do so, followed by radiotherapy and concomitant chemotherapy (12, 13).

The initial therapeutic approach for GBM is surgery, where maximal resection is associated with longer progression-free survival (PFS) and overall survival (OS) (14). Resection is not a curative approach; hence, patients typically undergo radiotherapy and chemotherapy as an adjunct (15). Radiotherapy, at a total dose of 60 Gy, is administered as either primary treatment or following surgery (16), both resulting in improvements to PFS and OS. Concomitant administration of temozolomide [150–200 mg/m^2^/day for 5 days each 28-days cycle (12, 13)], an oral alkylating agent, significantly increases OS in patients with newly diagnosed GBM from 12.1 months with radiotherapy alone to 14.6 months with radiotherapy and temozolomide (13). However, despite this increase in survival with radiotherapy and temozolomide, tumour progression and recurrence typically occur (17, 18), due to the development of resistance to temozolomide (19, 20). Once GBM recurrence occurs, therapeutic options for patients are limited (21, 22). Recently, tumour treating fields (TTFields; Optune), which deliver electric fields to the tumour location to disrupt cancer cell division, have emerged as an FDA-approved treatment for both recurrent and newly diagnosed GBM (23). However, the identification of new targets to facilitate the development of novel targeted therapies is warranted.

## Emerging Targeted Therapies

GBM is an invasive tumour with hallmarks of neoangiogenesis and intratumour heterogeneity, contributing to the poor prognosis observed (24). A variety of genetic and epigenetic alterations have been identified in GBM that influence patient prognosis (Table 1). Despite this heterogeneity, a large-scale analysis of genetic aberrations in GBM identified three main signalling pathways that are commonly dysregulated: activation of the receptor tyrosine kinase (RTK)/Ras/phosphoinositide 3-kinase (PI3K) pathway (88%), inhibition of p53 (87%), and retinoblastoma protein (Rb) signalling pathways (78%) (34). Drugs targeting many of these commonly observed alterations have been investigated as potential targeted therapies for GBM.

**Table 1:** Commonly identified genetic alterations in GBM.

**Name**	**Function**	**Expression status**	**Prevalence**	**Prognosis**	**References**
EPHA3	Regulation of adhesive and repulsive mechanisms including cell motility and adhesion	Overexpressed	40–60%	Poor; over-expression common in recurrent GBM	(25–27)
EGFR	Regulation of processes involved in cell growth, division and survival	Overexpressed	40–60%	Poor	(28–31)
MGMT	Prevention of mismatch errors	Methylated	40–60%	Favourable	(32, 33)
CDKN2A	Regulation of cell cycle and retinoblastoma activation	Decreased	49–52%	Poor	(34)
PTEN	Regulation of cell signalling. Involved in cell proliferation and survival	Deleted and/or mutated	34%	Poor	(29, 34–37)
PIK3CA	Regulation of processes involved in cell growth, division and survival	Overexpressed and/or mutated	15%	Poor; can predict recurrence	(34, 38, 39)
PDGFRA	Regulation of processes involved in cell growth, division and survival	Overexpressed	13%	Poor	(30, 34, 40, 41)
IDH1	Production of NADPH	Mutated	5–10%	Favourable	(42, 43)
MDM2	Regulation of p53 activity	Overexpressed	8–9%	Unclear	(44, 45)
MET	Regulation of proliferation, survival and motility	Overexpressed and/or mutated	4–6%	Poor	(34, 46–49)
SF/HGF	Activating ligand for HGFR/c-MET. Tumour growth and angiogenesis	Overexpressed	1.6–4%	Poor	(28, 47, 50)
VEGF	Promotion of angiogenesis	Overexpressed and/or mutated		Poor	(51–53)

*EPHA3, ephrin type-A receptor 3; EGFR, epidermal growth factor receptor; MGMT, O-6-methylguanine-DNA methyltransferase; CDKN2A, cyclin dependent kinase inhibitor 2A; PTEN, phosphatase and tensin homolog; PIK3CA, phosphatidylinositol-4,5-bisphosphate 3-kinase catalytic subunit alpha; PDGFRA, platelet derived growth factor receptor alpha; IDH1, isocitrate dehydrogenase 1; MDM2, double minute 2 protein; MET, hepatocyte growth factor receptor; SF/HGF, scatter factor/hepatocyte growth factor; VEGF, vascular endothelial growth factor*.

### EphA3 Receptor Inhibitors

The EphA3 receptor is overexpressed in 40–60% of GBM tumours and is commonly overexpressed in recurrent GBM (Table 1) (25). EphA3 is highly expressed on the tumour-initiating cell population in glioma, maintains tumour cells in a less differentiated and stem cell-like state, and its expression mediates the tumourigenic potential in GBM cells *in vitro* (26), suggesting that EphA3 may be a potential target for the treatment of GBM (Table 2).

**Table 2:** Targeted therapeutic outcomes in GBM.

**Target**	**Drug**	**Stage**	**Study design**	**Response**	**References**
EphA3	GLP1790	*In vitro*	T98G, A172, U251MG, U87MG monolayer, neurospheres and clonogenic assay	Decreased proliferation of cell cultures and cancer stem cells	(54)
		*In vivo*	U87MG, U251MG, T98G subcutaneous xenografts−30 mg/kg/day orally GLP1790, one dose of 4 Gy radiotherapy, temozolomide 16 mg/kg for 5 consecutive days, or radiotherapy + temozolomide	Increased survival *in vivo* compared to radiotherapy alone, but not temozolomide or radiotherapy + temozolomide	
		*In vivo*	U87MG intracranial xenografts−30 mg/kg/day orally GLP1790, one dose of 4 Gy radiotherapy, temozolomide 32 mg/kg for 5 consecutive days	Increased survival *in vivo* compared to radiotherapy alone, but not temozolomide or radiotherapy + temozolomide	
	IIIA4-USAN	*In vitro*	Four primary GBM samples	Decreased cell viability	(55)
		*In vivo*	U251MG, two GBM patient samples intracranial xenografts—(10 mg/kg, twice weekly intravenously)	Increased survival *in vivo* compared to unlabelled IIIA4 and vehicle treatment	
	EPHA2/A3 BsAb	*In vitro*	BT241, BT972 neurosphere and clonogenicity assays	Inhibits clonogenicit	(56)
		*In vivo*	BT241 intracranial xenografts—intracranial treatment twice a week with 9.4 μl EPHA2/A3 BsAB	Non-significant reduction in tumour burden compared to IgG control	
EGFR	Erlotinib	*In vitro*	Nine GBM cell lines	Inhibits anchorage-independent growth and induces apoptosis	(57)
		*In vitro*	060919 and 020919 (GBM oncosphere lines)	No inhibition of growth	(58)
		*In vitro*	Tumour initiating cells isolated from seven GBM patient samples	Time and dose-dependent inhibition of cell proliferation in all cultures (except GBM2)	(59)
		*In vivo*	Mayo39 and Mayo59 subcutaneous xenografts−40 mg/kg crizotinib daily by gavage, 100 mg/kg erlotinib daily by gavage, or crizotinib + erlotinib	Recues tumour burden	(60)
		*In vivo*	Primary GBM (GBM12 and GBM14) intracranial xenograft−100 mg/kg or 150 mg/kg daily erlotinib by gavage	Enhanced survival in wild-type PTEN and EGFR amplified tumours; Tumours lacking PTEN exhibit no survival benefit	(61)
		*Phase II*	Recurrent malignant glioma (*n = 53*) and non-progressive GBM (*n = 43*) following radiation therapy−150 mg/day erlotinib	Single agent activity was minimal for recurrent gliomas and marginally beneficial following radiotherapy for non-progressive GBM	(62)
		*Phase II*	Newly diagnosed GBM (*n = 65*)−100 mg/day erlotinib with radiotherapy and temozolomide and 150 mg/day after radiotherapy, with erlotinib dose escalated after radiotherapy until patients developed tolerable grade 2 rash or until maximum allowed dose reached	Improved survival times compared to historical controls	(63)
		*Phase II*	First relapse GBM (*n = 48*)−150 mg of erlotinib	Overall response rate of 6.3%, 6-months progression free survival of 20%, median survival of 9.7 months	(64)
	Gefitinib	*In vitro*	Tumour cell migration in GBM organotypic slice cultures	Decreased tumour cell migration in EGFR-amplified tumours	(65)
		*In vitro*	020913 and 060919(GBM oncosphere lines)	Slight growth inhibition with gefitinib alone, enhanced with the addition of sunitinib; block of oncosphere regrowth following gefitinib and sunitinib co-treatment	(58)
		*In vivo*	020913 intracranial xenograft−75 mg/kg gefitinib, 15 mg/kg sunitinib, or gefitinib + sunitinib	Gefitinib alone increased survival compared to vehicle and sunitinib treated; Addition of sunitinib did not further increase survival	
		*In vitro*	Tumour initiating cells isolated from seven GBM patient samples	Time and dose-dependent inhibition of cell proliferation in all cultures (except GBM2)	(59)
		*Phase II*	Recurrent glioblastoma (*n = 22*)−500 mg gefitinib for 5 days prior to surgery, followed by post-operative gefitinib until recurrence	Median survival after initiation of gefitinib treatment was 8.8 months; no difference between patients with amplified or normal EGFR.	(66)
		*Phase I/II*	Newly diagnosed glioblastoma patients (phase I *n = 31*; Phase II *n = 147*)—daily oral gefitinib commenced at the time of conventional cranial radiotherapy and continued post-radiotherapy for 18 months or until progression	No overall survival benefit of the addition of gefitinib when compared to historical cohort of patients treated with radiotherapy alone	(67)
		*Phase II*	Newly diagnosed GBM (*n = 96*)−500 mg/day gefitinib	Addition of gefitinib produced no survival benefit when compared to historical cohort of patients treated with radiotherapy alone	(68)
	Cetuximab	*In vitro*	Primary GBM (Ros57, Jon52, Mor56)	Induced apoptosis as a monotherapy and when combined with radiotherapy	(69)
		*In vivo*	Ros57 subcutaneous xenograft−0.5 mg cetuximab intraperitoneally twice per week for 5 weeks, or cetuximab + 2 or 4 Gy radiotherapy	Arrest tumour growth (depended on size of tumour at treatment commencement)	
		*In vivo*	Ros57 or Jon52 intracranial xenografts−0.5 mg cetuximab intraperitoneally twice weekly for duration	Increased survival	
		*In vitro*	U373MG, U87MG, Ros57, Jon52, Mor56, Bai, Roc	Induced apoptosis in EGFR-amplified lines	(70)
		*In vivo*	Ros57, Jon52 subcutaneous xenograft−0.5 or 1 mg cetuximab intraperitoneally twice weekly	Decreased tumour burden and increased survival, and eliminated Ros57 tumours	
		*Phase II*	Recurrent high-grade glioma (*n = 55*)−400 mg/m^2^ on week 1 cetuximab intravenously, followed by weekly dose of 250 mg/m^2^	Well-tolerated but limited activity	(71)
VEGF	Bevacizumab	*In vivo*	G55 intracranial xenograft−10–100 μg intraperitoneally twice weekly	Decreased tumour growth and vessel density	(72)
		*In vivo*	U87MG intracranial and intradermal xenograft−98.4 μg intraperitoneally every third day	Reduced vessel permeability and tumour volume	(73)
		*In vivo*	U87MG subcutaneous xenograft−100 μg intraperitoneally every second day, six doses combined with radiotherapy	Decrease in tumour burden when used as a monotherapy, and at least an additive increase when combined with radiotherapy	(74)
		*In vivo*	U87MG intracerebral xenograft−1 mg intraperitoneal every third day, three doses	Decreased tumour growth	(75)
		*Meta-analysis*	Four clinical trials (*n = 607*)	No difference in overall survival, modest increase in progression-free survival when combined with chemotherapy, compared with bevacizumab or chemotherapy alone. Higher incidence of treatment-related adverse events in bevacizumab treated patients.	(76)
	Tivozanib	*Phase II*	Recurrent glioblastoma (*n = 10*)−1.5 mg tivozanib daily, 3 weeks on/1 week off in 28-days cycles	Despite functional changes in tumour vasculature, limited anti-tumour activity was observed	(77)
	Pazopanib	*Phase II*	Recurrent glioblastoma (*n = 35*)−800 mg daily on 4-weeks cycles	Despite demonstrating biological activity (determined by radiographic responses), single-agent pazopanib did not prolong progression free survival	(78)
PDGFR	Imatinib	*In vitro*	U251MG and SF539 cell lines	Gleevec sensitised GBM cells to irradiation	(79)
		*In vivo*	GL261 intracranial xenograft model−3 mg of Gleevec by gavage on days 5, 7, and 9, 3 Gy radiotherapy on Days 5–9, or Gleevec + radiotherapy	Gleevec monotherapy improved survival at a level similar to radiotherapy, the combination of Gleevec and radiotherapy significantly enhanced survival	(80)
		*In vivo*	U87MG intracranial xenograft−50 mg/kg intraperitoneally	Increased survival	(81)
		*Phase II*	Recurrent GBM (*n = 51*)−800 mg/d with dose escalation to 1,000 mg/d	Well-tolerated but has limited anti-tumour activity	(82)
		*Phase III*	Progressive pre-treated GBM resistant to standard dose temozolomide (*n = 240*)−600 mg/d imatinib in combination with 1,000 mg/d of hydroxyurea, or 1,500 mg/d of hydroxyurea alone	The addition of imatinib did not increase progression free survival	(83)
	Sunitinib	*In vitro*	U87MG, GL15 cells implanted into organotypic brain slices	Sunitinib induced apoptosis and decreased proliferation	(84)
		*In vivo*	U87MG intracerebral xenograft model−80 mg/kg sunitinib orally, 5 days on, 2 days off	Improved median survival and reduced microvessel density	
		*In vivo*	PDGF-driven mouse model (PDGF-RES-Cre retrovirus infection of adult glial progenitors in mice carrying conditional deletions of PTEN and p53)−60 mg/kg sunitinib gavaged daily on a 5 day on, 2 days off cycle, 2 or 6 Gy radiotherapy, or a combination of both	Sunitinib or high-dose radiotherapy alone delayed tumour growth and increased survival. The addition of sunitinib to low-dose radiotherapy delayed tumour growth, with no survival benefit. Sunitinib combined with high dose radiotherapy induced a fatal toxicity.	(85)
		*Phase II*	Recurrent GBM (*n = 6*)−37.5 mg/day sunitinib for 14 days	Overall response rate was 17%, and 6-months progression free survival was not reach. Trial terminated due to insufficient activity.	(86)
		*Phase II*	First-line treatment of patients with GBM (*n = 47*)−2 Gy/day, 75 mg/m^2^ oral temozolomide daily, 6 months of maintenance temozolomide therapy with 150 mg/m^2^ oral on Days 1–5 every 28 days, and sorafenib (400 mg twice daily orally)	Addition of sorafenib did not improve progression free survival when compared with standard therapy	(87)
PI3K Pathway Inhibitors	Buparlisib	*In vitro*	U87MG	Decreases cell growth	(88)
		*In vivo*	U87MG subcutaneous xenograft−30 or 60 mg/kg Buparlisib orally daily	Decreased tumour growth	
		*In vitro*	U373MG, LNZ308, U251MG, SNB19, LN751, LN428, U87-V111, U87-E, U343, LN229, U251-E, D54, U-251-V111, A172, U87-PTEN-V, LN18, U87-PTEN-E, T98G, U87MG, SF767	Dose-dependent growth inhibition, and differential sensitivity pattern with respect to p53 status (wild-type p53 more sensitive than mutant or p53 null)	(89)
		*In vivo*	U87MG intracranial xenograft−20 or 40 mg/kg buparlisib once per day on a 5 days on, 2 days off schedule for 4 weeks	Increased survival	
		*Phase II*	Recurrent glioblastoma (*n = 50*)−100 mg daily	Minimal effect on progression free and overall survival	(90)
	Sonolisib	*In vitro*	U251MG, U87MG, LN229, LN18	Whilst sonolisib did not induce apoptosis, it inhibited invasion and angiogenesis	(91)
		*In vivo*	U87MG intracranial xenografts−2 mg/kg sonolisib orally on a 5 days on, 2 days off schedule for 4 weeks	Increased the median survival time	
		*In vitro*	U87MG, LNZ308, LN229	Combination with BBR3610 resulted in synergistic killing	(92)
			U87MG intracranial xenograft−0.1 mg/kg BBR3610 intravenously once a week for 3 weeks, 2 mg/kg sonolisib orally three times a week for a total of 12 treatment, or combination of BBR3610 and sonolisib	Enhanced survival time	
		*Phase II*	Recurrent GBM (*n = 33*)−8 mg of sonolisib daily in 8 weeks cycles	Overall response rate was low	(39)
HGFR/MET inhibitors	SGX532	*In vitro*	U87MG, U373MG, A172, DAOY, GBM10 and glioma stem cells 1228−30 nM−1.5 μM SGX532, 1 h	Inhibition of tumour growth, invasion and migration	(93)
		*In vivo*	U87MG intracranial xenograft−50 mg/kg SGX532 every 12 h for 3 weeks	Decreased tumour growth	(93)
	Amuvatinib (MP470)	*In vitro*	SF763, SF268, SF295, SF126, SF188, SF767, U87MG and SF210−5–10 μM MP470 1 h prior to irradiation	Enhanced radiosensitivity	(94)
	Crizotinib	*In vivo*	Mayo39 and Mayo59 intracranial xenografts−40 mg/kg crizotinib daily for 7 continuous days by gavage	Inhibition of tumour growth and depletion of sphere-forming cells	(95)
		*In vivo*	Mayo39 and Mayo59 subcutaneous xenografts−40 mg/kg crizotinib daily by gavage, 100 mg/kg erlotinib daily by gavage, or crizotinib + erlotinib	Reduced tumour burden and vascular density (when given in combination with erlotinib)	(60)

A small molecule inhibitor of the EphA3 receptor, GLPG1790, has demonstrated superior tumour reduction in U251MG and U87MG subcutaneous xenograft models when compared to radiotherapy alone, however, GLPG1790 was not as effective as treatment with radiotherapy and concomitant temozolomide (54). Whilst GLPG1790 did not exhibit improved benefit over the current therapies, additional strategies for targeting EphA3 are being examined. An EphA3 monoclonal antibody (IIIA4) that binds the EphA3 globular ephrin-binding domain has been developed, and the humanised version (ifabotuzumab) is the subject of an investigator-sponsored Phase 0/1 clinical trial currently underway in patients with recurrent GBM (55) to identify the optimal dose for tumour penetration. The IIIA4 antibody conjugated to the cytotoxic microtubule-targeting agent maytansine (IIIA4-USAN), induced apoptosis in four primary GBM cell lines *in vitro*, and significantly increased survival in an orthotopic model *in vivo* (55). Providing further evidence for the potential suitability of monoclonal antibodies targeting EphA3, a bispecific antibody against EphA2/A3 reduced clonogenicity *in vitro* and decreased tumour burden *in vivo* (56). Taken together, these studies indicate that EphA3 receptor inhibitors may be promising treatments for EphA3 receptor-amplified GBM, including recurrent disease, however, this remains to be tested in the clinic.

### EGFR Inhibitors

Epidermal Growth Factor Receptor (EGFR) amplifications and mutations are detected in 40–60% of GBM cases (28, 96) (Table 1) and are generally indicative of poor prognosis (97). EGFR (also referred to as ERBB1 or HER1) is a member of the HER superfamily of RTKs, along with ERBB2, ERBB3, and ERBB4. Binding of a ligand to the ligand-binding site of these receptors induces receptor homo- or heterodimerisation, producing a conformational change that activates the intracellular tyrosine kinase domain. This results in autophosphorylation of the cytoplasmic tail and induces a variety of downstream signalling pathways. The overexpression or mutation of EGFR leads to downstream signalling that impairs apoptosis, enhances proliferation, and angiogenesis. The most common mutant form found in GBM is ΔEGFR (EGFRvIII, or de2-7EGFR), arising through an 801 base pair in-frame deletion from the extracellular domain (98). Due to the high incidence of EGFR amplifications, a variety of EGFR inhibitors have been examined both pre-clinically and clinically (Table 2).

#### Small Molecule Inhibitors

Small molecule tyrosine kinase inhibitors are the most widely studied EGFR inhibitors in GBM, and include erlotinib, gefitinib, and lapatinib. Erlotinib inhibits anchorage-independent growth of GBM cells *in vitro* in an EGFR expression-dependent manner and induces greater levels of apoptosis in more malignant GBM phenotypes (57). The tumour-initiating cell population, which is resistant to radiotherapy (99), is sensitive to erlotinib in a phosphatase and tensin homolog (PTEN) and Akt dependent manner (59), suggesting that erlotinib may eliminate this population *in vivo*. Further, treatment with erlotinib was shown to reduce tumour burden in two GBM patient-derived xenograft (PDX) models (60). However, further studies using additional GBM PDX models demonstrated that tumours overexpressing EGFR were only sensitive to erlotinib if they also expressed PTEN (61). As PTEN expression is downregulated in ~34% of GBM patients (29), this indicates that erlotinib may not be a suitable treatment for the majority of GBM patients overexpressing EGFR. Indeed, erlotinib was not effective as a monotherapy in recurrent GBM patients and was only marginally beneficial following radiotherapy for non-progressive GBM patients (62). Despite a limited number of complete and partial responses in a Phase II study in first-relapse GBM, the 6-months PFS and median survival was similar to that previously reported for patients undergoing chemotherapy (64), suggesting that erlotinib may be useful in this setting. However, due to the non-randomised nature of this trial, these results must be interpreted cautiously. By contrast, improved survival (19.3 vs. 14.1 months) was observed when combined with temozolomide and radiotherapy (63), suggesting that erlotinib may be beneficial when combined with other treatments, rather than as a monotherapy.

In contrast to erlotinib, gefitinib exhibits anti-tumour activity independent of the expression level of EGFR (100). Gefitinib inhibits GBM cell migration (65), reduces proliferation of human glioma tumour-initiating cells *in vitro* (59) and enhances survival in an intracranial GBM mouse xenograft model *in vivo* (58). Taken together, these pre-clinical studies indicate that gefitinib may be clinically beneficial. However, despite gefitinib reaching high concentrations in GBM tumour tissue (22-fold higher compared to plasma) and the significant dephosphorylation of EGFR achieved (66), limited clinical effects have been observed in Phase II trials. Several Phase I/II studies have demonstrated that whilst the addition of gefitinib to radiotherapy is well-tolerated, it has no survival benefit (67, 68).

#### Monoclonal Antibodies

Although tumour immunotherapy has shown some success for the treatment of melanoma and haematological cancers, the applicability to GBM presents more of a challenge. Monoclonal antibodies directed against wild-type EGFR and ΔEGFR have been developed, with the best characterised in GBM being cetuximab. Pre-clinical studies have shown that treatment with cetuximab alone and in combination with radiotherapy increases survival *in vivo* (69) and can also completely eliminate tumours in EGFR-amplified PDX models (70). A phase II trial examining cetuximab treatment in patients with recurrent high-grade glioma showed that cetuximab was well-tolerated, but exhibited limited activity in this patient population (71).

### VEGF Inhibitors

With the largely disappointing clinical results for EGFR inhibitors, additional targets are being investigated, including vascular endothelial growth factor (VEGF), which is highly expressed in glioma cells. High VEGF expression is directly associated with the poor prognosis and malignancy of gliomas (51–53, 101). VEGF is a dimeric polypeptide that binds to the VEGF receptors 1 (VEGFR-1) and VEGFR-2, and the co-receptors neuropilin 1 and 2. Following this interaction, VEGF mediates angiogenesis and cell proliferation. Under hypoxic conditions, hypoxia-inducible transcription factors translocate to the nucleus which activate *VEGF* leading to increased angiogenesis in an attempt to counteract hypoxia (102). GBM tumours are commonly hypoxic and have increased VEGF expression that contributes to the irregular vasculature in GBM, prompting the investigation of VEGF as a potential therapeutic target (Table 2).

#### Small Molecule Inhibitors

Several VEGF inhibitors have been examined for the treatment of GBM, including the small molecule inhibitors, tivozanib, and pazopanib. Phase II studies of tivozanib (77) and pazopanib (78) in recurrent glioblastoma showed that these inhibitors exhibited limited anti-tumour activity and did not prolong PFS in this patient population. These trials highlight the limitations of anti-VEGF monotherapy.

#### Monoclonal Antibodies

Bevacizumab, a humanised monoclonal antibody against VEGF, blocks angiogenesis and thereby reduces tumour growth in a variety of GBM mouse models as a monotherapy and when combined with radiotherapy (72–75). These promising pre-clinical studies led to the clinical investigation and subsequent approval of bevacizumab for the treatment of recurrent GBM (103). However, a meta-analysis of four clinical trials including 607 patients demonstrated that the addition of bevacizumab to standard chemo-radiotherapy in the upfront setting only improves PFS, with no improvement in OS, but with an increase in the number of treatment-related adverse events (76). Of note, a decline in neurocognitive function is more frequently observed following bevacizumab treatment, as bevacizumab impairs hippocampal synaptic plasticity and decreases dendritic spine number and length (104). The modest treatment responses combined with the increased treatment-related adverse events raises concerns about the suitability of the use of bevacizumab as a treatment for GBM.

### Inhibition of Multiple RTKs

Multiple RTKs are coactivated in GBM tumours (105), introducing redundancy and limiting the efficacy of therapies targeting single RTKs. EGFR and platelet derived growth factor receptor A (PDGFRA) protein co-expression occurs in 37% of GBM (106). PDGFRA is the second most frequently amplified (10–13%) receptor tyrosine kinase in GBM (28, 107) (Table 1). A variety of multi-RTKs inhibitors have been examined both pre-clinically and clinically (Table 2).

Imatinib is a small molecule that inhibits PDGFRA and PDGFRB, as well as the RTKs c-Abl and c-Kit, and is a radiation-sensitising agent for glioma cells *in vitro* (79) and in orthotopic GBM models *in vivo* (80, 81). These promising pre-clinical studies led to the initiation of clinical trials. However, whilst imatinib was well-tolerated in recurrent GBM patients, it exhibited limited anti-tumour activity (82). Subsequent Phase III trials have examined imatinib in combination with hydroxyurea, rather than as a monotherapy (83). This trial showed that there was no PFS benefit to the addition of imatinib to hydroxyurea, or hydroxyurea alone. Further, a recent study has demonstrated that imatinib treatment can increase GBM cell migration and invasion *in vitro* (108), providing further potential insight as to why imatinib has failed in clinical trials for GBM.

Following the failure of imatinib in clinical trials, additional multi-RTK inhibitors have been studied. Sunitinib is an oral, small molecule that inhibits PDFR and VEGFR, and thereby reduces vascularisation and triggers apoptosis to produce tumour reduction. In pre-clinical models, sunitinib treatment induced apoptosis *in vitro*, and improved survival in an intracerebral GBM mouse model (84). Further, sunitinib treatment delayed tumour growth and increased survival in a PDGFF-driven mouse model, both as a monotherapy and in combination with low dose radiotherapy (85). By contrast, a Phase II trial and systematic review of the literature indicated that compared to conventional chemotherapy or bevacizumab, sunitinib has limited clinical activity in recurrent GBM as a monotherapy (86) or when combined with temozolomide or radiotherapy and temozolomide as a first-line treatment of patients with GBM (87).

### PI3K Pathway Inhibitors

Genetic aberrations in GBM, including *EGFR, PDGFRA, PTEN, TP53*, and *PIK3CA*, drive the dysfunction of signalling pathways, including PI3K/Akt/mTOR, p53, and Rb1 (34). The PI3K/Akt signalling pathway is activated in most GBMs and plays a critical role in the regulation of signal transduction, and mediates a variety of cellular processes, including proliferation, survival, migration and angiogenesis in GBM. The PI3K/Akt pathway is typically initiated via the activation of RTKs or G protein-coupled receptors, were the conformational changes in the C-terminal kinase domain produced by autophosphorylation provides binding sites for the regulatory subunits of PI3K, which leads to elevated lipid kinase activity of PI3K, and activation of Akt. Despite the limited clinical efficacy of the previously described RTK inhibitors, as activation of each of these receptors leads to downstream activation of the PI3K/Akt pathway, it has therefore been suggested that PI3K pathway inhibitors may be beneficial in GBM. More than 50 PI3K inhibitors have been designed and are under investigation as treatments for a range of cancers. Several PI3K inhibitors have demonstrated pre-clinical efficacy in GBM (Table 2) and have entered into clinical trials for GBM treatment.

Buparlisib, a pan PI3K inhibitor, reduces GBM cell growth both *in vitro* and *in vivo* (88, 89). Buparlisib is the most frequently used PI3K inhibitor in clinical trials for GBM treatment, as it is well-tolerated and BBB permeable. However, single agent efficacy in Phase II trials in recurrent glioblastoma has been minimal (90). The lack of clinical efficacy was explained by incomplete blockade of the PI3K pathway in the tumour tissue. Whilst buparlisib showed minimal single-agent efficacy, the study of other PI3K inhibitors that achieve more-complete pathway inhibition may still be warranted.

Sonolisib is an irreversible wortmannin analogue that demonstrates a more persistent inhibitor effect on PI3K than wortmannin. Sonolisib inhibits invasion and angiogenesis in GBM cell lines *in vitro* and extends survival benefit in orthotopic xenograft models *in vivo* (91, 92). Despite these promising pre-clinical results, the response rate to sonolisib in a Phase II study in patients with recurrent GBM was low, and the study did not meet its primary endpoint (109).

### HGFR/MET Inhibitors

Brain tumours secrete scatter factor (SF)/hepatocyte growth factor (HGF), the activating ligand for HGFR/MET (50), which has been associated with poor prognosis for GBM patients (Table 1) (110, 111). HGF is overexpressed in 1.6–4% of GBM patients, and via activation of MET, enhances tumour growth and angiogenesis (28). The *MET* proto-oncogene encodes for MET, an RTK that is overexpressed in 4–6% of GBM patients. A mutated, constitutively active variant of MET, MET^Δ7−8^, has been identified in 6% of patients with high grade gliomas, including GBM, enhancing downstream signalling to promote tumour progression and angiogenesis (46).

SGX-523 is a small molecule inhibitor of HGFR/MET tyrosine kinase activity that inhibits tumour cell growth, migration and invasion in a panel of glioma cells *in vitro* and reduced tumour growth in a murine xenograft model of GBM using the U87MG GBM cell line (Table 2) (93). Additionally, amuvatinib (MP470) is a small molecule inhibitor that acts on multiple tyrosine kinases, including MET, has been shown to radiosensitise GBM cell lines both *in vitro* and *in vivo* (Table 2) (94).

Another small molecule inhibitor of MET kinase activity, crizotinib, inhibits the growth, sphere-forming capacity and expression of stem cell markers in a subcutaneous xenograft model of GBM using the U87MG cell line (95). However, in a subcutaneous xenograft model using Mayo39 and Mayo59 GBM cell lines, crizotinib was only effective at reducing tumour burden and vascular density when used in combination with the EGFR inhibitor erlotinib (60).

## Concluding Remarks

GBM is an often-fatal disease and the standard treatment options available to patients are only minimally effective. There is a growing body of evidence suggesting that a personalised therapeutic approach for the stratification of GBM patients to novel treatment regimens is necessary if survival rates for GBM patients are to improve. Indeed, genetic profiling of GBM biopsies has revealed aberrant expression of several potential therapeutic targets, including a number of RTKs (EphA3, EGFR, VEGF, PDGFR, and MET), however, there has been varied and limited clinical success in the use of inhibitors of these targets as anti-cancer therapies. This highlights that a better understanding of the basic biology of GBM is required so that additional targets can be identified. Indeed, promising pre-clinical effects have been observed with EphA3 inhibitors, however, it remains to be seen whether this translates into the clinic. Whilst PI3K inhibitors have exhibited limited effects in clinical trials, they did not completely inhibit the PI3K pathway. Despite the promising outlook for personalised therapeutic approaches to treating GBM patients, the identification of therapeutics that can cross the BBB, whilst maintaining therapeutic concentrations, remains a challenge and is often not reported. Further, although targeted therapies show limited efficacy as single agents, the combination of several targeted therapies may be of benefit to GBM patients. Thus, additional research is urgently required to identify therapeutic targets in GBM and to design novel therapeutic strategies for the treatment of GBM.

## Author Contributions

OT and JB drafted the manuscript and reviewed the literature. OT, JB, and KS contributed to the preparation and editing of the manuscript. KS formulated the idea.

### Conflict of Interest

The authors declare that the research was conducted in the absence of any commercial or financial relationships that could be construed as a potential conflict of interest.

## References

[1] Jemal A Siegel R Xu J Ward E. Cancer statistics, 2010. CA Cancer J Clin. (2010) 60:277–300. 10.3322/caac.20073 20610543

[2] Iacob G Dinca EB. Current data and strategy in glioblastoma multiforme. J Med Life. (2009) 2:386–393. 20108752 PMC3019011

[3] Dobes M Khurana VG Shadbolt B Jain S Smith SF Smee R. Increasing incidence of glioblastoma multiforme and meningioma, and decreasing incidence of Schwannoma (2000–2008): findings of a multicenter Australian study. Surg Neurol Int. (2011) 2:176. 10.4103/2152-7806.90696 22276231 PMC3263004

[4] Ostrom QT Gittleman H Fulop J Liu M Blanda R Kromer C. CBTRUS statistical report: primary brain and central nervous system tumors diagnosed in the United States in 2008–2012. Neuro Oncol. (2015) 17(Suppl. 4):iv1–62. 10.1093/neuonc/nov189 26511214 PMC4623240

[5] Zhu P Du XL Lu G Zhu JJ. Survival benefit of glioblastoma patients after FDA approval of temozolomide concomitant with radiation and bevacizumab: a population-based study. Oncotarget. (2017) 8:44015–31. 10.18632/oncotarget.17054 28467795 PMC5546458

[6] Louis DN Perry A Reifenberger G von Deimling A Figarella-Branger D Cavenee WK. The 2016 World Health Organization classification of tumors of the central nervous system: a summary. Acta Neuropathol. (2016) 131:803–20. 10.1007/s00401-016-1545-1 27157931

[7] Kleihues P Ohgaki H. Primary and secondary glioblastomas: from concept to clinical diagnosis. Neuro Oncol. (1999) 1:44–51. 10.1093/neuonc/1.1.44 11550301 PMC1919466

[8] Ohgaki H Kleihues P. The definition of primary and secondary glioblastoma. Clin Cancer Res. (2013) 19:764–72. 10.1158/1078-0432.CCR-12-3002 23209033

[9] Kakee A Terasaki T Sugiyama Y. Brain efflux index as a novel method of analyzing efflux transport at the blood-brain barrier. J Pharmacol Exp Ther. (1996) 277:1550–9. 8667222

[10] Gan HK van den Bent M Lassman AB Reardon DA Scott AM. Antibody-drug conjugates in glioblastoma therapy: the right drugs to the right cells. Nat Rev Clin Oncol. (2017) 14:695–707. 10.1038/nrclinonc.2017.95 28675164

[11] Xu YY Gao P Sun Y Duan YR. Development of targeted therapies in treatment of glioblastoma. Cancer Biol Med. (2015) 12:223–37. 10.7497/j.issn.2095-3941.2015.0020 26487967 PMC4607828

[12] Davis ME. Glioblastoma: overview of disease and treatment. Clin J Oncol Nurs. (2016) 20:S2–8. 10.1188/16.CJON.S1.2-8 27668386 PMC5123811

[13] Stupp R Mason WP van den Bent MJ Weller M Fisher B Taphoorn MJ. Radiotherapy plus concomitant and adjuvant temozolomide for glioblastoma. N Engl J Med. (2005) 352:987–96. 10.1056/NEJMoa043330 15758009

[14] Allahdini F Amirjamshidi A Reza-Zarei M Abdollahi M. Evaluating the prognostic factors effective on the outcome of patients with glioblastoma multiformis: does maximal resection of the tumor lengthen the median survival? World Neurosurg. (2010) 73:128–34; discussion: e16. 10.1016/j.wneu.2009.06.001 20860940

[15] Vogelbaum MA. Does extent of resection of a glioblastoma matter? Clin Neurosurg. (2012) 59:79–81. 10.1227/NEU.0b013e31826b2e75 22960517

[16] Perkins A Liu G. Primary brain tumors in adults: diagnosis and treatment. Am Fam Physician. (2016) 93:211–7. 26926614

[17] Sathornsumetee S Rich JN. New approaches to primary brain tumor treatment. Anticancer Drugs. (2006) 17:1003–16. 10.1097/01.cad.0000231473.00030.1f 17001172

[18] Gallego O. Nonsurgical treatment of recurrent glioblastoma. Curr Oncol. (2015) 22:e273–81. 10.3747/co.22.2436 26300678 PMC4530825

[19] Happold C Roth P Wick W Schmidt N Florea AM Silginer M. Distinct molecular mechanisms of acquired resistance to temozolomide in glioblastoma cells. J Neurochem. (2012) 122:444–55. 10.1111/j.1471-4159.2012.07781.x 22564186

[20] Felsberg J Rapp M Loeser S Fimmers R Stummer W Goeppert M. Prognostic significance of molecular markers and extent of resection in primary glioblastoma patients. Clin Cancer Res. (2009) 15:6683–93. 10.1158/1078-0432.CCR-08-2801 19861461

[21] Osuka S Van Meir EG. Overcoming therapeutic resistance in glioblastoma: the way forward. J Clin Invest. (2017) 127:415–26. 10.1172/JCI89587 28145904 PMC5272196

[22] Weller M van den Bent M Tonn JC Stupp R Preusser M Cohen-Jonathan-Moyal E. European Association for Neuro-Oncology (EANO) guideline on the diagnosis and treatment of adult astrocytic and oligodendroglial gliomas. Lancet Oncol. (2017) 18:e315–29. 10.1016/S1470-2045(17)30194-8 28483413

[23] Branter J Basu S Smith S. Tumour treating fields in a combinational therapeutic approach. Oncotarget. (2018) 9:36631–44. 10.18632/oncotarget.26344 30564303 PMC6290966

[24] Yi Y Hsieh IY Huang X Li J Zhao W. Glioblastoma stem-like cells: characteristics, microenvironment, and therapy. Front Pharmacol. (2016) 7:477. 10.3389/fphar.2016.00477 28003805 PMC5141588

[25] Ferluga S Tomé CM Herpai DM D'Agostino R Debinski W. Simultaneous targeting of Eph receptors in glioblastoma. Oncotarget. (2016) 7:59860–76. 10.18632/oncotarget.10978 27494882 PMC5312354

[26] Day BW Stringer BW Al-Ejeh F Ting MJ Wilson J Ensbey KS. EphA3 maintains tumorigenicity and is a therapeutic target in glioblastoma multiforme. Cancer Cell. (2013) 23:238–48. 10.1016/j.ccr.2013.01.007 23410976

[27] Wykosky J Gibo DM Debinski W. A novel, potent, and specific ephrinA1-based cytotoxin against EphA2 receptor expressing tumor cells. Mol Cancer Ther. (2007) 6:3208–18. 10.1158/1535-7163.MCT-07-0200 18089715

[28] Brennan CW Verhaak RG McKenna A Campos B Noushmehr H Salama SR. The somatic genomic landscape of glioblastoma. Cell. (2013) 155:462–77. 10.1016/j.cell.2013.09.034 24120142 PMC3910500

[29] An Z Aksoy O Zheng T Fan QW Weiss WA. Epidermal growth factor receptor and EGFRvIII in glioblastoma: signaling pathways and targeted therapies. Oncogene. (2018) 37:1561–75. 10.1038/s41388-017-0045-7 29321659 PMC5860944

[30] Furnari FB Cloughesy TF Cavenee WK Mischel PS. Heterogeneity of epidermal growth factor receptor signalling networks in glioblastoma. Nat Rev Cancer. (2015) 15:302–10. 10.1038/nrc3918 25855404 PMC4875778

[31] Hu X Miao W Zou Y Zhang W Zhang Y Liu H. Expression of p53, epidermal growth factor receptor, Ki-67 and O6-methylguanine-DNA methyltransferase in human gliomas. Oncol Lett. (2013) 6:130–4. 10.3892/ol.2013.1317 23946790 PMC3742817

[32] Hegi ME Diserens AC Gorlia T Hamou MF de Tribolet N Weller M. MGMT gene silencing and benefit from temozolomide in glioblastoma. N Engl J Med. (2005) 352:997–1003. 10.1056/NEJMoa043331 15758010

[33] Shen D Liu T Lin Q Lu X Wang Q Lin F. MGMT promoter methylation correlates with an overall survival benefit in Chinese high-grade glioblastoma patients treated with radiotherapy and alkylating agent-based chemotherapy: a single-institution study. PLoS ONE. (2014) 9:e107558. 10.1371/journal.pone.0107558 25211033 PMC4161443

[34] The Cancer Genome Atlas Research Network (TCGA) Comprehensive genomic characterization defines human glioblastoma genes and core pathways. Nature. (2008) 455:1061–8. 10.1038/nature07385 18772890 PMC2671642

[35] Benitez JA Ma J D'Antonio M Boyer A Camargo MF Zanca C. PTEN regulates glioblastoma oncogenesis through chromatin-associated complexes of DAXX and histone H3.3. Nat Commun. (2017) 8:15223. 10.1038/ncomms15223 28497778 PMC5437297

[36] Zheng H Ying H Yan H Kimmelman AC Hiller DJ Chen AJ. p53 and Pten control neural and glioma stem/progenitor cell renewal and differentiation. Nature. (2008) 455:1129–33. 10.1038/nature07443 18948956 PMC4051433

[37] Walker C Baborie A Crooks D Wilkins S Jenkinson MD. Biology, genetics and imaging of glial cell tumours. Br J Radiol. (2011) 84:S90–106. 10.1259/bjr/23430927 22433833 PMC3473897

[38] Gallia GL Rand V Siu IM Eberhart CG James CD Marie SK. PIK3CA gene mutations in pediatric and adult glioblastoma multiforme. Mol Cancer Res. (2006) 4:709–14. 10.1158/1541-7786.MCR-06-0172 17050665

[39] Zhao HF Wang J Shao W Wu CP Chen ZP To ST. Recent advances in the use of PI3K inhibitors for glioblastoma multiforme: current preclinical and clinical development. Mol Cancer. (2017) 16:100. 10.1186/s12943-017-0670-3 28592260 PMC5463420

[40] Koschmann C Zamler D MacKay A Robinson D Wu YM Doherty R. Characterizing and targeting PDGFRA alterations in pediatric high-grade glioma. Oncotarget. (2016) 7:65696–06. 10.18632/oncotarget.11602 27582545 PMC5323185

[41] Cantanhede IG de Oliveira JRM. PDGF family expression in glioblastoma multiforme: data compilation from Ivy glioblastoma atlas project database. Sci Rep. (2017) 7:15271. 10.1038/s41598-017-15045-w 29127351 PMC5681588

[42] SongTao Q Lei Y Si G YanQing D HuiXia H XueLin Z. IDH mutations predict longer survival and response to temozolomide in secondary glioblastoma. Cancer Sci. (2012) 103:269–73. 10.1111/j.1349-7006.2011.02134.x 22034964

[43] Cohen AL Holmen SL Colman H. IDH1 and IDH2 mutations in gliomas. Curr Neurol Neurosci Rep. (2013) 13:345. 10.1007/s11910-013-0345-4 23532369 PMC4109985

[44] Verreault M Schmitt C Goldwirt L Pelton K Haidar S Levasseur C. Preclinical efficacy of the MDM2 inhibitor RG7112 in MDM2-amplified and TP53 wild-type glioblastomas. Clin Cancer Res. (2016) 22:1185–96. 10.1158/1078-0432.CCR-15-1015 26482041 PMC4842012

[45] Reifenberger G Liu L Ichimura K Schmidt EE Collins VP. Amplification and overexpression of the MDM2 gene in a subset of human malignant gliomas without p53 mutations. Cancer Res. (1993) 53:2736–9. 8504413

[46] Navis AC van Lith SA van Duijnhoven SM de Pooter M Yetkin-Arik B Wesseling P. Identification of a novel MET mutation in high-grade glioma resulting in an auto-active intracellular protein. Acta Neuropathol. (2015) 130:131–44. 10.1007/s00401-015-1420-5 25862637 PMC4469304

[47] Cruickshanks N Zhang Y Yuan F Pahuski M Gibert M Abounader R. Role and therapeutic targeting of the HGF/MET pathway in glioblastoma. Cancers. (2017) 9:E87. 10.3390/cancers9070087 28696366 PMC5532623

[48] Miyamoto M Ojima H Iwasaki M Shimizu H Kokubu A Hiraoka N. Prognostic significance of overexpression of c-Met oncoprotein in cholangiocarcinoma. Br J Cancer. (2011) 105:131–8. 10.1038/bjc.2011.199 21673683 PMC3137414

[49] Kwak Y Kim SI Park CK Paek SH Lee ST Park SH. C-MET overexpression and amplification in gliomas. Int J Clin Exp Pathol. (2015) 8:14932–8. 26823824 PMC4713610

[50] Abounader R Laterra J. Scatter factor/hepatocyte growth factor in brain tumor growth and angiogenesis. Neuro Oncol. (2005) 7:436–51. 10.1215/S1152851705000050 16212809 PMC1871724

[51] Das S Marsden PA. Angiogenesis in glioblastoma. N Engl J Med. (2013) 369:1561–3. 10.1056/NEJMcibr1309402 24131182 PMC5378489

[52] Plate KH Breier G Millauer B Ullrich A Risau W. Up-regulation of vascular endothelial growth factor and its cognate receptors in a rat glioma model of tumor angiogenesis. Cancer Res. (1993) 53:5822–7. 7694795

[53] Ma C Li Y Zhang X Zhao G Xu H. Levels of vascular endothelial growth factor and matrix metalloproteinase-9 proteins in patients with glioma. J Int Med Res. (2014) 42:198–204. 10.1177/0300060513481924 24398760

[54] Gravina GL Mancini A Colapietro A Delle Monache S Sferra R Vitale F. The small molecule ephrin receptor inhibitor, GLPG1790, reduces renewal capabilities of cancer stem cells, showing anti-tumour efficacy on preclinical glioblastoma models. Cancers. (2019) 11:E359. 10.3390/cancers11030359 30871240 PMC6468443

[55] Offenhäuser C Al-Ejeh F Puttick S Ensbey KS Bruce ZC Jamieson PR. EphA3 pay-loaded antibody therapeutics for the treatment of glioblastoma. Cancers. (2018) 10:E519. 10.3390/cancers10120519 30562956 PMC6316644

[56] Qazi MA Vora P Venugopal C Adams J Singh M Hu A. Cotargeting ephrin receptor tyrosine kinases A2 and A3 in cancer stem cells reduces growth of recurrent glioblastoma. Cancer Res. (2018) 78:5023–37. 10.1158/0008-5472.CAN-18-0267 29945963

[57] Halatsch ME Gehrke EE Vougioukas VI Bötefür IC A-Borhani F Efferth T. Inverse correlation of epidermal growth factor receptor messenger RNA induction and suppression of anchorage-independent growth by OSI-774, an epidermal growth factor receptor tyrosine kinase inhibitor, in glioblastoma multiforme cell lines. J Neurosurg. (2004) 100:523–33. 10.3171/jns.2004.100.3.0523 15035290

[58] Joshi AD Loilome W Siu IM Tyler B Gallia GL Riggins GJ. Evaluation of tyrosine kinase inhibitor combinations for glioblastoma therapy. PLoS ONE. (2012) 7:e44372. 10.1371/journal.pone.0044372 23056179 PMC3462750

[59] Griffero F Daga A Marubbi D Capra MC Melotti A Pattarozzi A. Different response of human glioma tumor-initiating cells to epidermal growth factor receptor kinase inhibitors. J Biol Chem. (2009) 284:7138–48. 10.1074/jbc.M807111200 19147502 PMC2652334

[60] Goodwin CR Rath P Oyinlade O Lopez H Mughal S Xia S. Crizotinib and erlotinib inhibits growth of c-Met+/EGFRvIII+ primary human glioblastoma xenografts. Clin Neurol Neurosurg. (2018) 171:26–33. 10.1016/j.clineuro.2018.02.041 29803091

[61] Sarkaria JN Yang L Grogan PT Kitange GJ Carlson BL Schroeder MA. Identification of molecular characteristics correlated with glioblastoma sensitivity to EGFR kinase inhibition through use of an intracranial xenograft test panel. Mol Cancer Ther. (2007) 6:1167–74. 10.1158/1535-7163.MCT-06-0691 17363510

[62] Raizer JJ Abrey LE Lassman AB Chang SM Lamborn KR Kuhn JG. A phase II trial of erlotinib in patients with recurrent malignant gliomas and nonprogressive glioblastoma multiforme postradiation therapy. Neuro Oncol. (2010) 12:95–103. 10.1093/neuonc/nop015 20150372 PMC2940554

[63] Prados MD Chang SM Butowski N DeBoer R Parvataneni R Carliner H Phase II study of erlotinib plus temozolomide during and after radiation therapy in patients with newly diagnosed glioblastoma multiforme or gliosarcoma. J Clin Oncol. (2009) 27:579–84. 10.1200/JCO.2008.18.9639 19075262 PMC2645859

[64] Yung WK Vredenburgh JJ Cloughesy TF Nghiemphu P Klencke B Gilbert MR. Safety and efficacy of erlotinib in first-relapse glioblastoma: a phase II open-label study. Neuro Oncol. (2010) 12:1061–70. 10.1093/neuonc/noq072 20615922 PMC3018931

[65] Parker JJ Dionne KR Massarwa R Klaassen M Foreman NK Niswander L. Gefitinib selectively inhibits tumor cell migration in EGFR-amplified human glioblastoma. Neuro Oncol. (2013) 15:1048–57. 10.1093/neuonc/not053 23749785 PMC3714155

[66] Hegi ME Diserens AC Bady P Kamoshima Y Kouwenhoven MC Delorenzi M. Pathway analysis of glioblastoma tissue after preoperative treatment with the EGFR tyrosine kinase inhibitor gefitinib–a phase II trial. Mol Cancer Ther. (2011) 10:1102–12. 10.1158/1535-7163.MCT-11-0048 21471286

[67] Chakravarti A Wang M Robins HI Lautenschlaeger T Curran WJ Brachman DG. RTOG 0211: a phase 1/2 study of radiation therapy with concurrent gefitinib for newly diagnosed glioblastoma patients. Int J Radiat Oncol Biol Phys. (2013) 85:1206–11. 10.1016/j.ijrobp.2012.10.008 23182702 PMC4199329

[68] Uhm JH Ballman KV Wu W Giannini C Krauss JC Buckner JC. Phase II evaluation of gefitinib in patients with newly diagnosed Grade 4 astrocytoma: Mayo/North Central Cancer Treatment Group study N0074. Int J Radiat Oncol Biol Phys. (2011) 80:347–53. 10.1016/j.ijrobp.2010.01.070 20510539 PMC5753591

[69] Eller JL Longo SL Kyle MM Bassano D Hicklin DJ Canute GW. Anti-epidermal growth factor receptor monoclonal antibody cetuximab augments radiation effects in glioblastoma multiforme *in vitro* and *in vivo*. Neurosurgery. (2005) 56:155–62. 10.1227/01.NEU.0000145865.25689.55 15617598

[70] Eller JL Longo SL Hicklin DJ Canute GW. Activity of anti-epidermal growth factor receptor monoclonal antibody C225 against glioblastoma multiforme. Neurosurgery. (2002) 51:1005–13; discussion: 1013–4. 10.1227/00006123-200210000-00028 12234411

[71] Neyns B Sadones J Joosens E Bouttens F Verbeke L Baurain JF. Stratified phase II trial of cetuximab in patients with recurrent high-grade glioma. Ann Oncol. (2009) 20:1596–603. 10.1093/annonc/mdp032 19491283

[72] Kim KJ Li B Winer J Armanini M Gillett N Phillips HS. Inhibition of vascular endothelial growth factor-induced angiogenesis suppresses tumour growth *in vivo*. Nature. (1993) 362:841–4. 10.1038/362841a0 7683111

[73] Yuan F Chen Y Dellian M Safabakhsh N Ferrara N Jain RK. Time-dependent vascular regression and permeability changes in established human tumor xenografts induced by an anti-vascular endothelial growth factor/vascular permeability factor antibody. Proc Natl Acad Sci USA. (1996) 93:14765–70. 10.1073/pnas.93.25.14765 8962129 PMC26210

[74] Lee CG Heijn M di Tomaso E Griffon-Etienne G Ancukiewicz M Koike C Anti-vascular endothelial growth factor treatment augments tumor radiation response under normoxic or hypoxic conditions. Cancer Res. (2000) 60:5565–70. 11034104

[75] Gossmann A Helbich TH Kuriyama N Ostrowitzki S Roberts TP Shames DM. Dynamic contrast-enhanced magnetic resonance imaging as a surrogate marker of tumor response to anti-angiogenic therapy in a xenograft model of glioblastoma multiforme. J Magn Reson Imaging. (2002) 15:233–240. 10.1002/jmri.10072 11891967

[76] Yang SB Gao KD Jiang T Cheng SJ Li WB. Bevacizumab combined with chemotherapy for glioblastoma: a meta-analysis of randomized controlled trials. Oncotarget. (2017) 8:57337–44. 10.18632/oncotarget.16924 28915674 PMC5593645

[77] Kalpathy-Cramer J Chandra V Da X Ou Y Emblem KE Muzikansky A. Phase II study of tivozanib, an oral VEGFR inhibitor, in patients with recurrent glioblastoma. J Neurooncol. (2017) 131:603–10. 10.1007/s11060-016-2332-5 27853960 PMC7672995

[78] Iwamoto FM Lamborn KR Robins HI Mehta MP Chang SM Butowski NA. Phase II trial of pazopanib (GW786034), an oral multi-targeted angiogenesis inhibitor, for adults with recurrent glioblastoma (North American Brain Tumor Consortium Study 06-02). Neuro Oncol. (2010) 12:855–61. 10.1093/neuonc/noq025 20200024 PMC2940686

[79] Russell JS Brady K Burgan WE Cerra MA Oswald KA Camphausen K. Gleevec-mediated inhibition of Rad51 expression and enhancement of tumor cell radiosensitivity. Cancer Res. (2003) 63:7377–83. 14612536

[80] Geng L Shinohara ET Kim D Tan J Osusky K Shyr Y. STI571 (Gleevec) improves tumor growth delay and survival in irradiated mouse models of glioblastoma. Int J Radiat Oncol Biol Phys. (2006) 64:263–71. 10.1016/j.ijrobp.2005.08.025 16274936

[81] Kilic T Alberta JA Zdunek PR Acar M Iannarelli P O'Reilly T. Intracranial inhibition of platelet-derived growth factor-mediated glioblastoma cell growth by an orally active kinase inhibitor of the 2-phenylaminopyrimidine class. Cancer Res. (2000) 60:5143–50. 11016641

[82] Raymond E Brandes AA Dittrich C Fumoleau P Coudert B Clement PMJ. Phase II study of imatinib in patients with recurrent gliomas of various histologies: a European Organisation for Research and Treatment of Cancer Brain Tumor Group Study. J Clin Oncol. (2008) 26:4659–65. 10.1200/JCO.2008.16.9235 18824712 PMC2653126

[83] Dresemann G Weller M Rosenthal MA Wedding U Wagner W Engel E. Imatinib in combination with hydroxyurea versus hydroxyurea alone as oral therapy in patients with progressive pretreated glioblastoma resistant to standard dose temozolomide. J Neurooncol. (2010) 96:393–402. 10.1007/s11060-009-9976-3 19688297

[84] de Boüard S Herlin P Christensen JG Lemoisson E Gauduchon P Raymond E Antiangiogenic and anti-invasive effects of sunitinib on experimental human glioblastoma. Neuro Oncol. (2007) 9:412–23. 10.1215/15228517-2007-024 17622648 PMC1994098

[85] D'Amico R Lei L Kennedy BC Sisti J Ebiana V Crisman C. The addition of Sunitinib to radiation delays tumor growth in a murine model of glioblastoma. Neurol Res. (2012) 34:252–61. 10.1179/1743132812Y.0000000005 22449730 PMC3812673

[86] Grisanti S Ferrari VD Buglione M Agazzi GM Liserre R Poliani L. Second line treatment of recurrent glioblastoma with sunitinib: results of a phase II study and systematic review of literature. J Neurosurg Sci. (2019) 63:458–67. 10.23736/S0390-5616.16.03874-1 27680966

[87] Hainsworth JD Ervin T Friedman E Priego V Murphy PB Clark BL. Concurrent radiotherapy and temozolomide followed by temozolomide and sorafenib in the first-line treatment of patients with glioblastoma multiforme. Cancer. (2010) 116:3663–9. 10.1002/cncr.25275 20564147

[88] Burger MT Pecchi S Wagman A Ni ZJ Knapp M Hendrickson T. Identification of NVP-BKM120 as a potent, selective, orally bioavailable class I PI3 kinase inhibitor for treating cancer. ACS Med Chem Lett. (2011) 2:774–9. 10.1021/ml200156t 24900266 PMC4017971

[89] Koul D Fu J Shen R LaFortune TA Wang S Tiao N. Antitumor activity of NVP-BKM120-a selective pan class I PI3 kinase inhibitor showed differential forms of cell death based on p53 status of glioma cells. Clin Cancer Res. (2012) 18:184–95. 10.1158/1078-0432.CCR-11-1558 22065080 PMC3785365

[90] Wen PY Touat M Alexander BM Mellinghoff IK Ramkissoon S McCluskey CS. Buparlisib in patients with recurrent glioblastoma harboring phosphatidylinositol 3-kinase pathway activation: an open-label, multicenter, multi-arm, phase II trial. J Clin Oncol. (2019) 37:741–50. 10.1200/JCO.18.01207 30715997 PMC6553812

[91] Koul D Shen R Kim YW Kondo Y Lu Y Bankson J. Cellular and *in vivo* activity of a novel PI3K inhibitor, PX-866, against human glioblastoma. Neuro Oncol. (2010) 12:559–69. 10.1093/neuonc/nop058 20156803 PMC2940638

[92] Gwak HS Shingu T Chumbalkar V Hwang YH DeJournett R Latha K. Combined action of the dinuclear platinum compound BBR3610 with the PI3-K inhibitor PX-866 in glioblastoma. Int J Cancer. (2011) 128:787–96. 10.1002/ijc.25394 20473884 PMC2990813

[93] Guessous F Zhang Y diPierro C Marcinkiewicz L Sarkaria J Schiff D. An orally bioavailable c-Met kinase inhibitor potently inhibits brain tumor malignancy and growth. Anticancer Agents Med Chem. (2010) 10:28–35. 10.2174/1871520611009010028 20015006 PMC3278215

[94] Welsh JW Mahadevan D Ellsworth R Cooke L Bearss D Stea B. The c-Met receptor tyrosine kinase inhibitor MP470 radiosensitizes glioblastoma cells. Radiat Oncol. (2009) 4:69. 10.1186/1748-717X-4-69 20028557 PMC2806296

[95] Rath P Lal B Ajala O Li Y Xia S Kim J. *In vivo* c-met pathway inhibition depletes human glioma xenografts of tumor-propagating stem-like cells. Transl Oncol. (2013) 6:104–11. 10.1593/tlo.13127 23556031 PMC3612837

[96] Li J Di C Mattox AK Wu L Adamson DC. The future role of personalized medicine in the treatment of glioblastoma multiforme. Pharmgenomics Pers Med. (2010) 3:111–27. 10.2147/PGPM.S6852 23226047 PMC3513213

[97] Li J Liang R Song C Xiang Y Liu Y. Prognostic significance of epidermal growth factor receptor expression in glioma patients. Onco Targets Ther. (2018) 11:731–42. 10.2147/OTT.S155160 29445288 PMC5808691

[98] Gan HK Kaye AH Luwor RB. The EGFRvIII variant in glioblastoma multiforme. J Clin Neurosci. (2009) 16:748–54. 10.1016/j.jocn.2008.12.005 19324552

[99] Bao S Wu Q McLendon RE Hao Y Shi Q Hjelmeland AB. Glioma stem cells promote radioresistance by preferential activation of the DNA damage response. Nature. (2006) 444:756–60. 10.1038/nature05236 17051156

[100] Mellinghoff IK Wang MY Vivanco I Haas-Kogan DA Zhu S Dia EQ. Molecular determinants of the response of glioblastomas to EGFR kinase inhibitors. N Engl J Med. (2005) 353:2012–24. 10.1056/NEJMoa051918 16282176

[101] Li Q Qiao G Ma J Li Y. Downregulation of VEGF expression attenuates malignant biological behavior of C6 glioma stem cells. Int J Oncol. (2014) 44:1581–8. 10.3892/ijo.2014.2331 24627040

[102] Shibuya M. Structure and function of VEGF/VEGF-receptor system involved in angiogenesis. Cell Struct Funct. (2001) 26:25–35. 10.1247/csf.26.25 11345501

[103] Cohen MH Shen YL Keegan P Pazdur R. FDA drug approval summary: bevacizumab (Avastin) as treatment of recurrent glioblastoma multiforme. Oncologist. (2009) 14:1131–8. 10.1634/theoncologist.2009-0121 19897538

[104] Latzer P Shchyglo O Hartl T Matschke V Schlegel U Manahan-Vaughan D. Blocking VEGF by bevacizumab compromises electrophysiological and morphological properties of hippocampal neurons. Front Cell Neurosci. (2019) 13:113. 10.3389/fncel.2019.00113 30971896 PMC6445260

[105] Stommel JM Kimmelman AC Ying H Nabioullin R Ponugoti AH Wiedemeyer R. Coactivation of receptor tyrosine kinases affects the response of tumor cells to targeted therapies. Science. (2007) 318:287–90. 10.1126/science.1142946 17872411

[106] Chakravarty D Pedraza AM Cotari J Liu AH Punko D Kokroo A. EGFR and PDGFRA co-expression and heterodimerization in glioblastoma tumor sphere lines. Sci Rep. (2017) 7:9043. 10.1038/s41598-017-08940-9 28831081 PMC5567352

[107] Nazarenko I Hede SM He X Hedrén A Thompson J Lindström MS. PDGF and PDGF receptors in glioma. Ups J Med Sci. (2012) 117:99–112. 10.3109/03009734.2012.665097 22509804 PMC3339542

[108] Frolov A Evans IM Li N Sidlauskas K Paliashvili K Lockwood N. Imatinib and Nilotinib increase glioblastoma cell invasion via Abl-independent stimulation of p130Cas and FAK signalling. Sci Rep. (2016) 6:27378. 10.1038/srep27378 27293031 PMC4904410

[109] Pitz MW Eisenhauer EA MacNeil MV Thiessen B Easaw JC Macdonald DR. Phase II study of PX-866 in recurrent glioblastoma. Neuro Oncol. (2015) 17:1270–4. 10.1093/neuonc/nou365 25605819 PMC4588751

[110] Kong DS Song SY Kim DH Joo KM Yoo JS Koh JS. Prognostic significance of c-Met expression in glioblastomas. Cancer. (2009) 115:140–8. 10.1002/cncr.23972 18973197

[111] Petterson SA Dahlrot RH Hermansen SK K A Munthe S Gundesen MT Wohlleben H. High levels of c-Met is associated with poor prognosis in glioblastoma. J Neurooncol. (2015) 122:517–27. 10.1007/s11060-015-1723-3 25800004

